# Engineering of Saposin C Protein Chimeras for Enhanced Cytotoxicity and Optimized Liposome Binding Capability

**DOI:** 10.3390/pharmaceutics13040583

**Published:** 2021-04-19

**Authors:** Suzanne I. Sandin, David M. Gravano, Christopher J. Randolph, Meenakshi Sharma, Eva de Alba

**Affiliations:** 1 Department of Bioengineering, University of California, Merced, CA 95343, USA; ssandin@ucmerced.edu (S.I.S.); crandolph@ucmerced.edu (C.J.R.); msharma32@ucmerced.edu (M.S.); 2 Chemistry and Chemical Biology Ph.D. Program, University of California, Merced, CA 95343, USA; 3 Stem Cell Instrumentation Foundry, University of California, Merced, CA 95343, USA; dgravano@ucmerced.edu

**Keywords:** proteoliposomes, saposin C, PUMA, liposome fusion, glioblastoma

## Abstract

Saposin C (sapC) is a lysosomal, peripheral-membrane protein displaying liposome fusogenic capabilities. Proteoliposomes of sapC and phosphatidylserine have been shown to be toxic for cancer cells and are currently on clinical trial to treat glioblastoma. As proof-of-concept, we show two strategies to enhance the applications of sapC proteoliposomes: (1) Engineering chimeras composed of sapC to modulate proteoliposome function; (2) Engineering sapC to modify its lipid binding capabilities. In the chimera design, sapC is linked to a cell death-inducing peptide: the BH3 domain of the Bcl-2 protein PUMA. We show by solution NMR and dynamic light scattering that the chimera is functional at the molecular level by fusing liposomes and by interacting with prosurvival Bcl-xL, which is PUMA’s known mechanism to induce cell death. Furthermore, sapC-PUMA proteoliposomes enhance cytotoxicity in glioblastoma cells compared to sapC. Finally, the sapC domain of the chimera has been engineered to optimize liposome binding at pH close to physiological values as protein–lipid interactions are favored at acidic pH in the native protein. Altogether, our results indicate that the properties of sapC proteoliposomes can be modified by engineering the protein surface and by the addition of small peptides as fusion constructs.

## 1. Introduction

**Figure 1:**
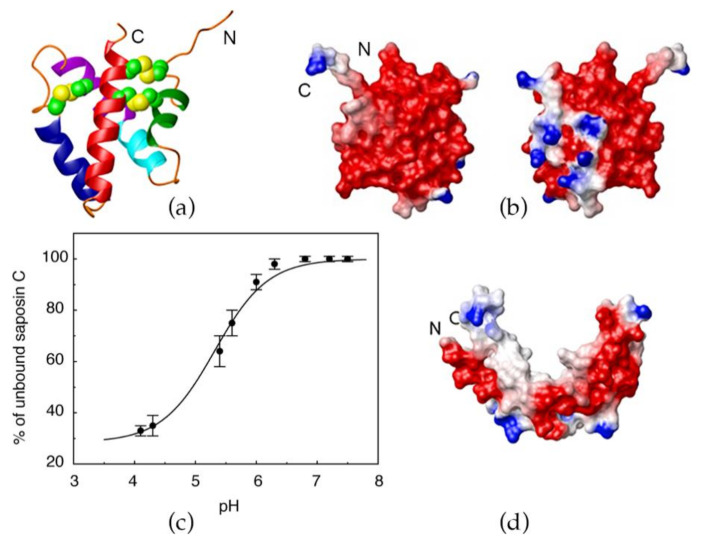
SapC binding to liposomes is a pH-dependent reversible process: (**a**) Ribbon diagram of the 3D NMR structure of sapC [9]; (**b**) Electrostatic surface charge distribution of sapC; (**c**) SapC reversible binding to liposomes with pH; (**d**) 3D structure of sapC bound to micelles [10]. (**a**–**c**): Reprinted from *Biochemistry* **2003**, *42*, 14729–14740. Published 2003 American Chemical Society; (**d**): Reprinted from *J. Mol. Biol.* **2005**, *346*, 1381–1392.

The protease-resistant, heat-stable, lysosomal protein saposin C (sapC), is a peripheral-membrane protein with important roles in lipid degradation [1] and lipid antigen presentation to CD1 (cluster of differentiation) proteins [2,3,4,5,6]. Importantly, sapC has been reported to produce liposome fusion at acidic pH and to show binding preference for negatively charged lipids such as phosphatidylserine (PS) [7,8]. A mechanistic investigation based on the three-dimensional (3D) solution NMR structure of sapC [9] in the absence and presence of micelles [10], and liposome binding studies under different conditions, explains the pH-dependence of sapC binding to liposomes. In the absence of lipids or micelles, sapC adopts the five-helix bundle motif with two pairs of helices connected by three disulfide bonds characteristic of the saposin fold (Figure 1a) [9]. The electrostatic surface of sapC is mainly negatively charged at neutral pH by the presence of abundant Glu and Asp amino acids (Figure 1b). SapC binds to lipids at acidic pH and detaches from the bilayer at neutral pH in a reversible process (Figure 1c) [9]. This behavior is explained by the neutralization at low pH of the negative charge of acidic residues at the protein surface, which otherwise precludes the protein from forming stable interactions with the hydrophobic tails of the lipids [9]. The 3D structure of sapC in the presence of micelles conserves the secondary structure of the saposin fold, but the tertiary structure opens, exposing the hydrophobic core to detergent molecules (Figure 1d) [10].

SapC shows special characteristics, such as high stability and protease resistance, liposome fusogenic activity and pH-dependent liposome binding. In addition, sapC can be over-expressed by recombinant methods and purified by standard chromatographic techniques. Altogether, these attributes point to sapC as an attractive candidate for biotechnological applications and biologics design. In fact, proteoliposomes formed by sapC and liposomes of phospholipid 1,2-dioleoyl-sn-glycero-3-phosphatidylserine (DOPS) have been shown to promote apoptosis in cancer cells [11,12]. It has been suggested that sapC attached to the liposome lipid bilayer in the proteoliposomes [12] can recognize cell populations with increased content in PS lipids due to its preference to bind negatively charged lipids [13]. It has also been indicated that PS is more abundant in the plasma membrane of cancer cells than in healthy cells [14], which would explain the targeting of sapC proteoliposomes for specific cell populations [13]. As a result of these studies, sapC-DOPS proteoliposomes are currently undergoing clinical trial to treat refractory glioblastoma [11].

With the aim of enhancing the properties and applications of sapC proteoliposomes, we have designed as proof-of-concept a protein chimera carrying sapC at the N-terminus and the active domain (BH3 domain) of the Bcl-2 proapoptotic protein PUMA at the C-terminus. Hence, we refer to the chimera as sapC-PUMA. In addition, we have modified the electrostatic surface of sapC in the chimera to improve liposome binding for potential applications at physiological pH.

PUMA is an intrinsically disordered protein of the BH3-only subfamily that promotes apoptosis by antagonizing prosurvival Bcl-2 members [15]. The known molecular mechanism that PUMA follows to induce apoptosis consists in direct binding to antiapoptotic Bcl-xL [16]. It has been shown that the ~25 amino acid-region spanning PUMA’s BH3 domain (PUMA^BH3^) suffices for promoting cell death. BH3-derived peptides and peptidomimetics are intensively investigated as anticancer drugs [17,18]. Some BH3 domains are specific for certain prosurvival proteins; however, proapoptotic PUMA^BH3^ is promiscuous and potently binds with high affinity multiple prosurvival Bcl-2 proteins [19]. Because of these special characteristics, PUMA^BH3^ is an ideal candidate for a peptide-based anticancer drug.

We have characterized at the structural and functional levels both chimeras under cell-free conditions and in cells. We show by solution NMR that the structure of sapC in the sapC-PUMA chimera is not perturbed by the presence of the PUMA^BH3^ peptide, and the chimera is still capable of binding liposomes under mildly acidic conditions (pH 6.0). Analogously, our NMR data show that PUMA^BH3^ in the chimera is capable of binding Bcl-xL. The binding of sapC to liposomes is low at pH 7 (Figure 1c). This behavior is not optimal for applications that require the protein to be attached to the liposome outer leaflet at physiological pH, although the protein can be encapsulated and attached to the inner leaflet. With the purpose of enhancing sapC-PUMA binding to liposomes at neutral pH, we have designed and studied a mutant of the chimera by substituting two acidic residues in the sapC domain by positively charged amino acids (sapC-PUMA-DM). We show that liposome binding increases at pH 7 for the mutant chimera compared to the wildtype, thus pointing to a suitable strategy for additional modifications to enhance binding at neutral pH. However, the design of the double mutant is not intended to affect cytotoxicity of sapC-liposome particles in cancer cells. We further show, using dynamic light scattering, that both sapC-PUMA and sapC-PUMA-DM induce liposome fusion, which indicates that the saposin fold tolerates non-conservative mutations and still retains its fusogenic capability in the presence of the C-terminal PUMA^BH3^ fragment. Moreover, proteoliposomes with sapC-PUMA and sapC-PUMA-DM show increased cytotoxicity in glioblastoma cells relative to sapC-only proteoliposomes, which proves that the presence of PUMA^BH3^ has an additive effect in reducing cell viability. These chimera proteins are a proof-of-concept that saposin C can be engineered to modify its lipid-binding affinity and to potentiate cytotoxicity towards cancer cells. However, different functionalities could be envisioned for sapC chimeras in the presence or absence of liposomes by attaching different peptide/protein fragments and by modifying sapC’s electrostatic surface.

## 2. Materials and Methods

### 2.1. Protein Expression and Purification

The DNA sequences of sapC-PUMA and sapC-PUMA-DM were inserted in pET-30b vectors containing a thrombin cleavage site followed by His-tag at the C-terminus of PUMA^BH3^. Plasmids were created by Gene Universal Inc. (Newark, DE, USA) and the DNA sequencing results were double-cheked to be correct. Plasmids were transformed into *Escherichia coli* BL21(DE3) cells and grown in ^15^N-enriched minimal media for NMR studies and in Luria Broth for all other experiments. Protein expression was induced using 1 mM isopropyl β-D-thiogalactopyranoside (IPTG) at 37 °C for 4 h. Cells were harvested by centrifugation at 8 k rpm for 30 min and resuspended in 20 mM Tris (pH 8.0), 20 mM imidazole, 500 mM NaCl and phenylmethylsulfonyl fluoride (PMSF) as a protease inhibitor. Cells were broken by sonication at 20 kHz for 48 min on ice with on-off cycles of 15 s and 45 s, respectively. The sonicated solution was centrifuged at 35 k rpm for 45 min. The cell lysate was filtered through a 0.45 µm pore filter. Proteins were purified by HPLC using Ni^2+^ affinity chromatography with an elution gradient from 20 mM to 500 mM imidazole. Subsequently, proteins were dialyzed against a buffer containing 20 mM Tris (pH 8.0), 150 mM NaCl, 2.5 mM CaCl_2_, and subjected to thrombin cleavage overnight. For certain 1D-^1^H NMR experiments (vide infra), some protein samples were not cleaved to retain the His-tag. In all cases, the proteins were further purified by reverse phase chromatography using a C4 column (Higgings Analytical, Mountain View, CA, USA) in water and acetonitrile mixtures with 0.1% TFA. Protein solutions eluted from reverse phase were lyophilized. The solid protein was then resuspended in HPLC-grade water for different experiments. Human Bcl-xL-ΔTM (native amino acid sequence without the transmembrane domain) was cloned into the pET21a vector with a N-terminal six-His tag and was purified using the same methods described for sapC-PUMA constructs, with the addition of 500 µM tris (2-carboxyethyl) phosphine (TCEP) in the resuspension and elution buffers for Ni^2+^ affinity chromatography. Bcl-xL-ΔTM was not subjected to dialysis or thrombin cleavage before reverse phase chromatography.

### 2.2. Liposome Preparation for Cell-Free Assays

Brain L-α-phosphatidylserine lipids in chloroform were purchased from Avanti Polar Lipids. Lipids were dried overnight in vacuum and resuspended in water to a lipid concentration of 2 mM. Lipids were allowed to hydrate for 1 h, vortexed for 10 min, and sonicated by bath sonication for 20 min. Ice was added to the bath sonicator to control the temperature during sonication. Liposome size was determined using dynamic light scattering.

For TEM preparations, liposomes were produced by drying under vacuum a solution of 10 mg/ml of lipids in chloroform. The dried lipid film was resuspended in 20 mM acetate buffer (pH 4.3) to a final lipid concentration of 1 mM. After resuspension, the sample was vortexed and bath sonicated to produce liposomes. The liposomes were subjected to 10 cycles of extrusion through a polycarbonate filter with a pore size of 100 nm.

Other liposome preparations for size exclusion chromatography and dynamic light scattering experiments include the hydrating and vortexing steps mentioned above with the following modifications: (1) Use of tip sonication at 4 kHz for 6 min (30 s on-off cycles) on ice; (2) Use of tip sonication as in (1) followed by extrusion through either 100 nm or 200 nm pore filter; (3) Same as (2) with the addition of 10 freeze–thaw cycles following the first extrusion set, and a second 20-time extrusion step after the freeze–thaw cycle. Freeze–thaw cycles include 3 min in dry ice bath with ethanol, followed by 3 min in 50 °C water bath. Data for these preparations are not shown.

We found that liposomes that were tip sonicated or bath sonicated were consistent in size with a diameter of approximately ~100 nm. Liposomes that underwent freeze–thaw cycles, whether they were extruded through 100 nm or 200 nm pore filter, resulted in an average diameter close to 190 nm based on dynamic light scattering data.

### 2.3. Proteoliposome Preparation for Cell Assays

All proteoliposome stocks were prepared at ~250 µM protein and 1.25 mM PS lipid concentrations (by weight). Protein-only and liposome-only controls were prepared at the same respective concentrations. The protocol for proteoliposome preparation was adapted from reference [12]. In summary, PS lipids were dried overnight under vacuum and the lyophilized protein was added to the dried lipid film. The lipid–protein mixture was hydrated in 20 mM citrate buffer at pH 5. PBS buffer was added subsequently to bring the final pH of the proteoliposomes to 7. The final concentration of protein stocks was determined by absorbance at 280 nm for sapC-PUMA and sapC-PUMA-DM as these proteins contain a Trp residue in the PUMA^BH3^ sequence. However, sapC concentration cannot be accurately determined by absorbance due to the lack of Trp amino acid. NMR spectra of sapC-PUMA and sapC were acquired twice for each protein and overlaid to determine the concentration of sapC based on that of sapC-PUMA using NMR signal intensity of amino acids Glu49 and Cys75 from the sapC region, which are isolated. Two protein stocks were prepared for cell assays resulting in final protein concentrations: 8 µM, 16 µM, 24 µM, 28 µM, 32 µM, and 40 µM for killing activity comparison of sapC and sapC-PUMA; and 10 µM, 20 µM, 30 µM, 35 µM, 40 µM, and 50 µM for killing activity comparison of sapC-PUMA and sapC-PUMA-DM.

### 2.4. Solubility Studies with pH

Solubility studies were conducted using an Agilent Cary 60 UV–vis spectrophotometer (Agilent Technologies, Santa Clara, CA, USA) at 280 nm. Prior to each measurement, the protein sample was centrifuged, and the supernatant was filtered through a 0.22 μm filter. The cuvette was washed between measurements and a blank measurement was run to ensure no residual protein material remained attached to the walls. SapC-PUMA and sapC-PUMA-DM solutions at 100 μM were initially brought at neutral pH and the pH was subsequently decreased by incremental addition of aliquots of dilute HCl solutions. The solubility tests were repeated twice. Data were analyzed using QtGrace software (Qt Group, Helsinki, Finland). The solubility of sapC was not studied by absorbance because of the absence of Trp residues.

### 2.5. NMR Spectroscopy

NMR experiments were acquired on a Bruker Avance III 600 MHz spectrometer (Bruker, Billerica, MA, USA) equipped with a cryoprobe. All samples were prepared at 10% D_2_O, 90% HPLC-grade H_2_O. 2D-NMR data were collected at 298 K using [^1^H-^15^N]-SOFAST-HMQC experiments [20]. Amide ^1^H-^15^N chemical shift assignments for sapC in the sapC-PUMA chimera were obtained using previously published assignment of sapC [9]. The spectra of sapC-PUMA had to be shifted in the ^1^H and ^15^N dimensions relative to sapC due to differences in the temperature calibration of the different probes used and to differences in the calibration of the basic frequencies of ^1^H and ^15^N of the different spectrometers. PUMA^BH3^ was not assigned, except for the NH pair in the indol group of the Trp side chain. Data were processed with TOPSPIN and NMRPipe software [21] and analyzed with SPARKY [22].

### 2.6. NMR Sample Preparation for SapC-PUMA and Liposome Binding Experiments

Initial liposome binding studies were done using 1D-^1^H NMR in unlabeled sapC-PUMA constructs with and without the His-tag prepared to a final protein concentration of 100 µM at pH 6.8 and 100 µM lipid concentration of liposomes. Samples with and without liposomes were subjected to pH adjustments using dilute solutions of HCl and NaOH in the pH range from 6.8 to 4.2. All samples were inserted into clean NMR tubes to avoid minor pH changes. 1D-^1^H NMR was used to confirm liposome binding at acidic pH for constructs without His-tag. Constructs with His-tag showed extensive line broadening at acidic pH in the absence of liposomes indicating significant protein aggregation (Figure S1, Supplementary Materials). Thus, all experiments for liposome titration used samples without the His-tag.

Liposome titrations were done using [^1^H-^15^N]-SOFAST-HMQC [20] experiments on ^15^N-labeled sapC-PUMA and sapC-PUMA-DM samples. Stock solutions were prepared in HPLC-grade water at pH 6.0 and 500 µm protein concentration, confirmed by absorbance at 280 nm. The final concentration of sapC-PUMA used for NMR experiments was 100 μM at pH 6.0. ^15^N-labeled sapC-PUMA constructs were mixed with increasing lipid concentration of PS liposomes at pH 6.0. Independent experiments were performed twice with freshly diluted protein and liposome samples. The first 1D projections of the 2D NMR experiments were used to monitor decrease in protein NMR signal intensity during the liposome titration. All 1D projections were baseline-corrected from 11 ppm to 4.7 ppm. The overall signal intensity was obtained by integration in the range from 9.3 ppm to 6.6 ppm using TOPSPIN software. The signal intensity from the spectrum acquired in the absence of lipids was assigned a value of 1 corresponding to 0% binding. The binding percentage for the spectra acquired in the presence of increasing concentrations of lipids is thus relative to this intensity. The binding isotherms resulting from plotting the binding percentage values versus liposome concentration were fitted to the Hill equation (*vide infra*) using the program Grace

$$\left[\theta \right]=\frac{{\left[L_{t}\right]}^{n}}{{\left[L_{t}\right]}^{n}+{\;K}_{D\;}}$$

where θ is the normalized binding, [L_t_] is the total concentration of liposomes, K_D_ is the dissociation constant for protein-liposome binding and n is the Hill coefficient indicating the number of adsorbed molecules per binding site (see below additional details on the fitting rationale).

The liposome concentration values used in the titration were calculated based on the average number of lipid molecules per liposome and the lipid molar concentration used. This number was calculated by determining the surface area of the outer and inner leaflets based on the average size of liposomes determined by dynamic light scattering (139 nm), assuming a lipid bilayer thickness of 4 nm, and an average lipid head group surface of 0.64 nm^2^ [23].

### 2.7. NMR Sample Preparation for SapC-PUMA and Bcl-xL-ΔTM Titration Experiments

Unlabeled Bcl-xL-ΔTM was dissolved at a concentration of 900 μM at pH 6.8 for titration experiments. ^15^N-labeled sapC-PUMA (and sapC-PUMA-DM) was mixed with unlabeled Bcl-xL-ΔTM to a final protein concentration of 100 μM in all experiments with increasing molar ratios of BCL-xL-ΔTM. The following sapC-PUMA:BCL-xL-ΔTM molar ratios were tested: 1:0, 1:0.5, 1:1, and 1:2. Intensity decays of NMR signals of sapC-PUMA (and sapC-PUMA-DM) upon complexation were obtained with SPARKY [22].

### 2.8. Liposome Fusion by Dynamic Light Scattering

Dynamic light scattering studies were conducted using a Malvern Panalytical Zetasizer Pro (Malvern Panalytical, Malvern, United Kingdom). The refractive index of liposome and all protein preparations was determined to be 1.3322 and 1.3327, respectively using an Abbe Mark III refractometer (Reichert, Buffalo, NY, USA). Absorbance values for protein and liposomes samples obtained at the wavelength of the zetasizer laser (632.8 nm) were 0.05. Stock solutions of sapC, sapC-PUMA, and sapC-PUMA-DM were filtered through 50 nm pore filters and liposome stocks were tested to ensure monodisperse populations. The final concentration of protein and lipids in the mixture was 100 µM each. SapC solution and liposomes were prepared at pH 4.2 and mixed to perform the dynamic light scattering experiments at pH 4.2 either in water or in buffer (10 mM sodium acetate and 150 mM NaCl). SapC-PUMA and sapC-PUMA-DM solutions, and liposomes were prepared at pH 6.0 and adjusted to pH 5.3. The protein-liposome mixtures were immediately centrifuged at 14 K rpm for one minute to remove any possible particles that could affect the dynamic light scattering results. After centrifugation, the solution was carefully transferred to a cuvette. The solution was allowed to equilibrate for 2 min before taking three measurements at each time point. Measurements were obtained every 20 min for a total of ~200 min. The intensity of the scattered light at each particle size was averaged for the three measurements and plotted using QtGrace.

### 2.9. Size Exclusion Chromatography

Sephacryl S-1000 superfine resin was packed in two stacked Tricorn 10/300 columns (GE Healthcare, Chicago, IL, USA) for a final volume of 51 ml. The column assembly was equilibrated using 10 mM HEPES, 150 mM NaCl, pH 7.4. Liposomes were prepared in the equilibration buffer and were injected onto the column and monitored using the following wavelengths: 240 nm, 260 nm, 280 nm, and 300 nm.

### 2.10. Transmission Electron Microscopy

All micrographs were obtained on a JEOL JEM 2010 transmission electron microscope (JEOL, Peabody, MA, USA) with a LaB_6_ filament at 200 kV. Liposomes at 1 mM lipid concentration were prepared in 20 mM acetate buffer at pH 4.2. A total of 4 µl of these samples were deposited onto 300 mesh carbon-coated copper grids. The solution was left to dry completely or for 20 min and was washed three times in 40 µl droplets of 1% PTA (phosphotungstic acid) negative staining solution at pH 7. Grids were stained for 5 min before wiping dry. Images were taken using a Gatan camera of 1350 × 1040 pixels.

### 2.11. Cell Viability Assays

U-87 MG glioblastoma cells were purchased from American Type Culture Collection (ATCC, Manassas, VA, USA). Cells were cultured in Eagle’s minimal essential medium (ATCC) supplemented with 10% heat-inactivated fetal bovine serum (FBS) (Life Technologies, Carlsbad, CA, USA) and 100 units/ml Penicillin-Streptomycin (Life Technologies). Primary human astrocytes and Astrocyte Medium were purchased from ScienCell Research Laboratories (Carlsbad, CA, USA) and cultured according to vendor instructions. Cells were cultured at 37 °C with 5% CO_2_ in a humidified incubator.

Eight hours prior to proteoliposome treatment, cells were plated at 10,000 cells per well in 100 µl of complete medium in a flat-bottom 96-well tissue culture plate. A volume of 25 µl of proteoliposome was added to each well and incubated for 72 h. Cells were harvested by collecting supernatants containing floating cells and lifting adherent cells with 0.05% Trypsin/EDTA (Life Technologies). Centrifugation of cells was performed at 400× *g* for 5 min. Both adherent and floating fractions were pooled and washed with FACS buffer (1X PBS + 2% FBS) and stained with the LIVE/DEAD Viability/Cytotoxicity Kit (Invitrogen, Carlsbad, CA, USA). Calcein AM and Ethidium homodimer-1 were used at final concentrations of 25 nM and 2 µM, respectively, in FACS buffer. Cells were incubated in the staining solution for 15 min at room temperature prior to analyzing by flow cytometry using a BD LSR II flow cytometer with 488 nm laser excitation and 530/30 (green) and 610/20 (red) bandpass emission filters. All analysis was performed using FlowJo v10 (BD Biosciences, San Jose, CA, USA) and Prism v6 (GraphPad, San Diego, CA, USA) software.

## 3. Results

### 3.1. Design of Protein Chimeras and Protein Solubility Tests

**Figure 2:**
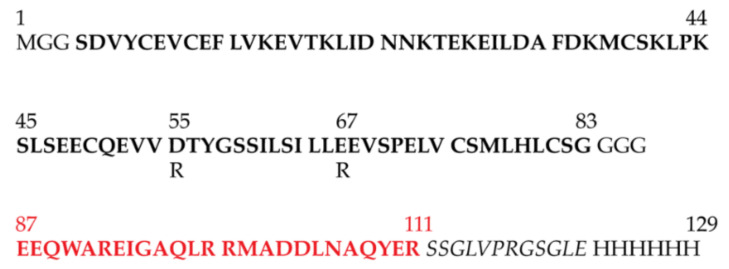
Amino acid sequence of engineered protein chimera sapC-PUMA and double-mutant sapC-PUMA-DM: The amino acid sequences (one letter code) of sapC (native sequence from residues S4 to G83) and PUMA^BH3^ (residues E87 to R111) are shown in bold black and bold red, respectively. Mutations in the sequence of sapC-PUMA-DM (D55R, E67R) are indicated. The thrombin cleavage site is in italics followed by the six-histidine tag.

The sequence of the sapC-PUMA chimera is shown in Figure 2. Human sapC is an 80 amino acid-long protein to which a short linker of three Gly amino acids is attached at the C-terminus to connect 25 additional amino acids encompassing PUMA^BH3^. The few extra amino acids at the C-terminus of the chimera include the His-tag for purification by affinity liquid chromatography, following a thrombin cleavage site to subsequently remove the His-tag.

Previous studies on a conservative mutant of sapC (Glu 9 Gln, Glu 12 Gln; in the chimera numbering as shown in Figure 2) designed to remove two negative charges from the protein surface, indicate that the mutant binds to liposomes to a greater extent under identical conditions as compared to wildtype sapC [9]. Based on this information, and with the aim of increasing liposome binding of the sapC-PUMA chimera at neutral pH, we designed a non-conservative double-mutant (sapC-PUMA-DM) that replaces two negative charges by positive charges: Asp 55 and Glu 67 were mutated to Arg (Figure 2). These mutations were chosen because of their location relatively separated from the contact region of sapC to the lipid bilayer based on the structure shown in Figure 1d [10] but still capable of influencing lipid binding.

**Figure 3:**
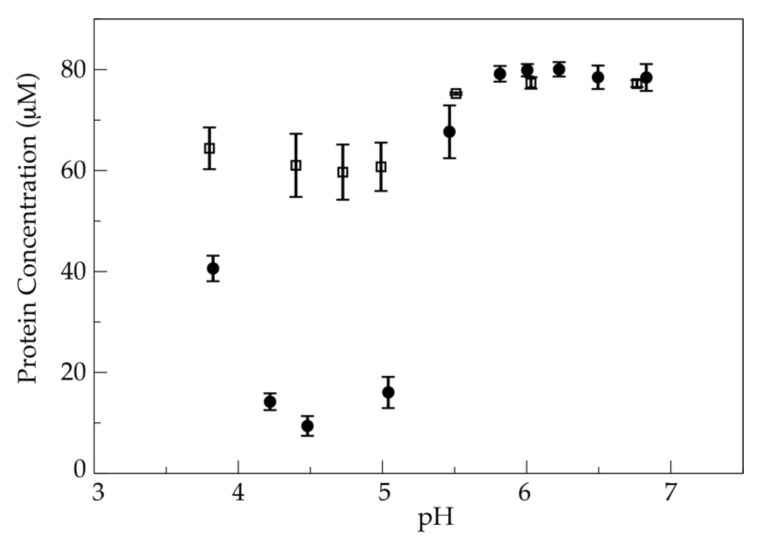
Solubility of the chimera increases by engineering the electrostatic surface of sapC: Average values of the solubility of sapC-PUMA (circles) and sapC-PUMA-DM (squares) measured by absorbance spectroscopy at 280 nm versus pH. Bars indicate the standard error associated to the repetition of the solubility experiments.

Because the binding of sapC to liposomes is pH-dependent, we studied the solubility of the different chimeras in a broad pH range to identify the best compromise between liposome binding and protein solubility. This information will be necessary to design different applications of sapC chimeras/liposome assemblies. Our results indicate that the isoelectric point of each chimera increases as the number of mutations to positively charged amino acids increases, as expected, and we observe that the overall solubility of the mutant chimera also increases (Figure 3).

### 3.2. Function of the SapC Domain in the Chimera Construct

#### 3.2.1. Structure of SapC Is not Perturbed in the SapC-PUMA Chimera

**Figure 4:**
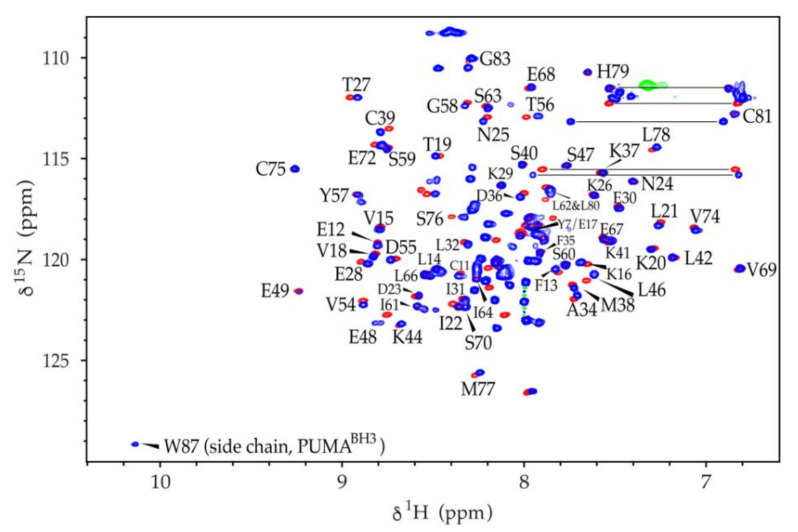
SapC overall structure is retained in sapC-PUMA: [^1^H-^15^N]-HSQC NMR spectra of sapC (red) and [^1^H-^15^N]-sofast-HMQC of sapC-PUMA (blue). Labels indicate amino acids in sapC-PUMA that do not show significant changes in chemical shifts. The NH signal of the Trp indole ring in PUMA is labeled. Several side chain NH_2_ signals of Asn and Gln residues are connected with a horizontal line.

To test whether the presence of PUMA in the sapC-PUMA chimera affects sapC at the structural level, we acquired [^1^H-^15^N]-2D NMR spectra of the chimera to compare with wildtype sapC. This type of experiment is known in protein NMR as the ‘protein fingerprint’, because it will be significantly perturbed upon small conformational or structural changes in the protein. The NMR data indicate a high degree of overlap between the ^1^H-^15^N amide signals of wildtype sapC and sapC in the chimera, which allowed almost the full assignment of the amide ^1^H and ^15^N chemical shifts of the sapC domain in the chimera (Figure 4). As expected, the [^1^H-^15^N]-2D spectrum of sapC-PUMA shows additional signals corresponding to the linker and PUMA^BH3^ (Figure 4). These results indicate that the structure of sapC within the engineered protein is unperturbed.

**Figure 5:**
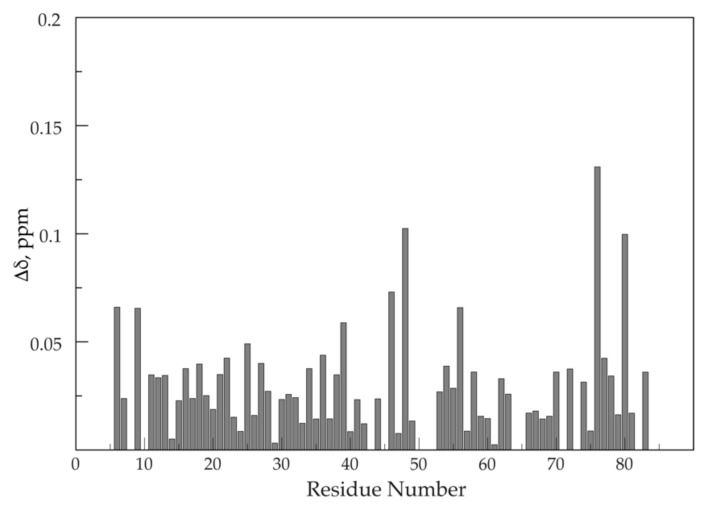
Small chemical shift differences between wilt-type sapC and sapC domain of sapC-PUMA: Combined amide ^1^H and ^15^N chemical shift differences of sapC and sapC-PUMA versus amino acid sequence.

The combined amide ^15^N and ^1^H chemical shift deviations between sapC and sapC-PUMA versus the amino acid sequence are shown in Figure 5. Amino acids with side chains that can be protonated and deprotonated by slight changes in the solution pH might show larger deviations in amide chemical shifts due to the effect of the protonation state of the side chain (Figure 5). For example, Glu 48, is one of the signals with the largest chemical shift changes. Amino acids Pro 43 and Pro 71 are not observed because they lack the NH amide pair that gives rise to signal in [^1^H-^15^N]-2D NMR spectra. Several amino acids such as Gln 51, Glu 52, Ile 64, and Leu 65, could not be unambiguously assigned and are not shown. Altogether, the average chemical shift change observed is small (0.03 ± 0.02 ppm), indicating that conformational or structural changes in sapC by the presence of the PUMA peptide are negligible.

#### 3.2.2. SapC in the Chimera Protein Is Capable of Binding Liposomes

Previously, we showed that sapC is capable of binding liposomes composed of phosphatidyl serine (PS) and phosphatidyl choline (PC) [9]. The binding at 1:1 protein/lipid molar ratio is negligible at neutral pH but increases at more acidic pH following a sigmoidal behavior, which reaches a plateau with binding close to 70% at pH 4.2 (Figure 1c) [9].

Based on the absence of structural changes in the sapC domain of the chimera, it is reasonable to expect that the function of sapC is also unmodified. To prove this hypothesis, we performed liposome binding assays with sapC-PUMA using NMR. For a better comparison to our previous work [9,10], these 2D NMR studies were performed under very similar conditions (absence of buffer for readily pH change to modulate sapC-liposome binding). However, sapC-PUMA titrations with liposomes were performed at pH 6.0, because of the higher solubility of the chimera at less acidic pH, and at increasing concentrations of PS liposomes due to the known preference of sapC for negatively charged lipids [7].

**Figure 6:**
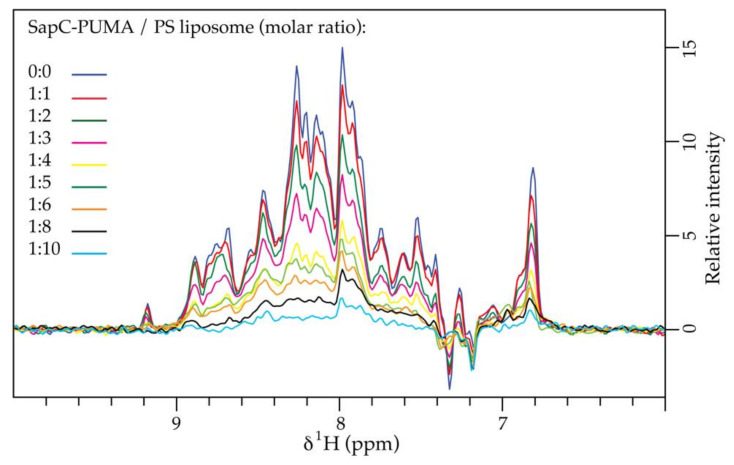
SapC-PUMA binds to liposomes at pH 6: 1D projections of [^1^H-^15^N]-sofast-HMQC experiments on chimera-liposome mixtures at increasing lipid concentration, showing the decrease in NMR signal intensity due to protein-liposome binding.

One-dimensional projections of [^1^H-^15^N]-sofast-HMQC experiments [20] on the chimera-liposome mixtures show a significant decrease in the NMR signal intensity at increasing lipid concentration (Figure 6). This result is analogous to the previously observed binding of wildtype sapC to PS/PC liposomes [9]. Liposome large size (~100 nm) results in an overall particle tumbling rate that is significantly slower compared to the free protein (with a rotational correlation time of 4.6 ns) [9], which impacts NMR signal to noise ratio due to magnetic relaxation processes. In fact, very large particles are ‘invisible’ in solution NMR. Thus, the overall NMR signal intensity decreases when the protein binds to liposomes because the effective population of protein free in solution decreases (Figure 6). Signal intensity loss is not accompanied by peak broadening; thus, intensity decrease does not result from changes in the magnetic relaxation properties of the protein that could reflect self-association or unfolding.

### 3.3. Binding of SapC-PUMA to Liposomes Can Be Tuned by Modifying the Electrostatic Surface of SapC

**Figure 7:**
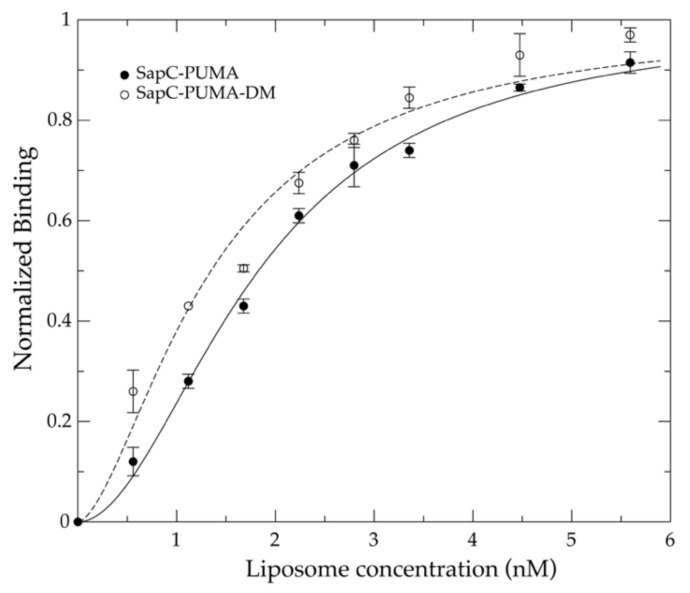
SapC-PUMA affinity for liposomes at pH 6 is increased by modifying the electrostatic surface of sapC: Fractional binding of sapC-PUMA and sapC-PUMA-DM to liposomes are shown in filled and open circles, respectively. Independent experiments were done twice, and the corresponding error bars indicate S.D. values. Liposome titrations were done using [^1^H-^15^N]-SOFAST-HMQC experiments on ^15^N-labeled sapC-PUMA and sapC-PUMA-DM samples that were mixed with increasing lipid concentration of PS liposomes at pH 6.0. The binding isotherms were fitted to the Hill equation as detailed in the experimental section.

The decrease in protein NMR signal intensity in the liposome titration follows a binding isotherm that cannot be properly fitted to the Langmuir equation for adsorption of gases in solid surfaces, most likely because the binding process does not satisfy the Langmuir conditions [24] (e.g., the adsorbed solutes do not interact with one another in a way that can modify the adsorption process). In fact, it is believed that saposin C dimerizes prior or during lipid binding [10], thus rendering the binding process more complex. The Langmuir’s theory is analogous to the Hill equation by considering the binding of ligands to a finite number of sites in a protein [25]. Thus, due to the law of mass action, both processes result in almost identical mathematical expressions with the possibility to include cooperative adsorption with the Hill equation. We have used the Hill equation to interpret the binding of sapC-PUMA to liposomes at pH 6 (Figure 7) and have obtained an apparent dissociation constant (K_Dapp_) value of ~3 nM and a Hill coefficient ~2, which indicates the number of adsorbed molecules per binding site in agreement with the dimerization mechanism.

The mutant chimera (sapC-PUMA-DM) in which the electrostatic surface of the sapC domain is more positively charged due to mutations of Asp 55 and Glu 67 to Arg, is expected to bind with increased affinity to liposomes. We first checked using [^1^H-^15^N]-2D NMR that the saposin fold was not perturbed by the presence of the mutations in the sapC domain. Figure S2 (Supplementary Materials) shows an overlay of the spectra of sapC-PUMA and sapC-PUMA-DM, indicating minimal perturbations of the chemical shifts and a properly folded protein. The NMR titration of sapC-PUMA-DM with liposomes indicates that liposome binding is enhanced in the presence of additional positive charges in the surface of sapC, resulting in a value of K_Dapp_ ~1.6 nM at pH 6. Thus, the affinity of sapC-PUMA-DM for liposomes has almost doubled relative to wildtype sapC-PUMA (Figure 7).

We also tested the binding of the different chimeras in mixtures at 1:10 protein/lipid molar ratio at pH 7 for potential applications in which sapC-PUMA needs to be attached to a significant extent to the liposome outer leaflet at physiological pH. As expected, binding is low for the wildtype chimera, showing a percentage of protein bound to liposomes of 8.5% from NMR data, whereas it increases to 28% for sapC-PUMA-DM (Figure S3, Supplementary Materials). These data indicate that amino acids that are positively charged in the surface of sapC facilitate liposome binding at physiological pH.

Our results could be important for sapC proteoliposomes aimed at targeting cancer cells [12]. The intravenous injection (pH ~ 7.5) of these assemblies will result in partial removal of wildtype sapC from the outer leaflet of the liposome, thus compromising to a certain extent the recognition of specific cell populations. An increase in the number of positive charges in the surface of sapC, such as in the double-mutant sapC-PUMA-DM, will help in increasing the presence of protein molecules attached to the outer leaflet of the liposome at physiological pH, thus potentially enhancing cell targeting. Altogether, our results indicate that liposome binding can be tuned by modifying the electrostatic surface of sapC via non-conservative mutations, which could result in different applications of the protein/liposome assembly.

### 3.4. Function of the PUMA Domain in the Chimera Constructs

PUMA is a BH3-only protein (containing the Bcl-2 homology domain #3) of the Bcl-2 family that promotes apoptosis or programmed cell death [19]. Pro- and anti-apoptotic members of the Bcl-2 family participate in protein–protein interactions as a mechanism to regulate cell fate [26,27]. It is well known that the BH3 region is the active binding component of the proapoptotic proteins [28,29]. Thus, peptides comprising the BH3 domain are capable of binding and eliciting cell death [15]. In addition, it has been extensively reported that certain types of cancer cells are resistant to anticancer treatments due to mechanisms developed to impair apoptosis [30]. Therefore, BH3-derived peptides from proapoptotic members of the Bcl-2 family are intensively investigated as anticancer drugs [31]. The BH3 domain of PUMA is capable of binding the prosurvival protein Bcl-xL as a mechanism of antagonizing its function and thus promoting cell death [19]. However, PUMA is unique in that it is the only protein capable of destabilizing the interaction between Bcl-xL and the transcription factor p53 to induce cell death [32]. Once free, p53 activates proapoptotic Bax and Bak, which trigger apoptosis by mitochondrial outer membrane permeabilization [32]. The structure of the complex between PUMA^BH3^ and Bcl-xL has been determined by NMR and the K_D_ of the interaction is ~3 nM based on isothermal titration calorimetry [16].

**Figure 8:**
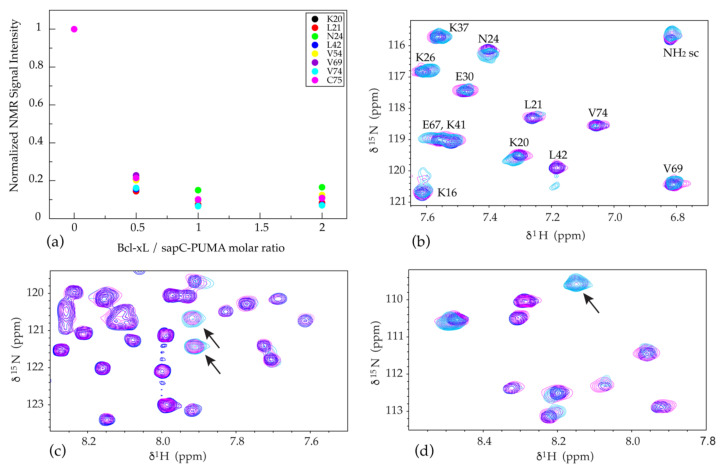
SapC-PUMA binds Bcl-xL via PUMA^BH3^ domain: (**a**) Decrease of NMR signal intensity for selected amino acids (indicated in the inset) of the sapC domain in sapC-PUMA as Bcl-xL/sapC-PUMA molar ratio increases. (**b**–**d**) Selected regions of overlaid [^1^H, ^15^N]-2D NMR spectra of ^15^N-labeled sapC-PUMA at Bcl-xL/sapC-PUMA molar ratios; 0:1 (blue), 0.5:1 (magenta) and 1:1 (cyan). SapC signals labeled with the corresponding amino acids are minimally perturbed in (**b**). New signals for the PUMA domain, indicated with arrows in (**c**,**d**) are observed at Bcl-xL/sapC-PUMA molar ratios 0.5:1 and 1:1.

To test the functionality of the PUMA^BH3^ component of the chimera, we have performed NMR titration experiments on ^15^N-labeled sapC-PUMA with unlabeled Bcl-xL at three different molar ratios (sapC-PUMA/Bcl-xL; 1:0.5, 1:1, 1:2; Figure 8). The binding of sapC-PUMA to Bcl-xL is apparent from the overall decrease in signal intensity and disappearance of numerous signals in the spectrum of the complexed chimera compared to free sapC-PUMA. The decrease in signal intensity as the concentration of Bcl-xL increases is shown for some amino acids of the sapC domain in Figure 8a. This result can be explained by the increase in molecular weight (slower tumbling rate) once sapC-PUMA (~14.4 kDa) binds Bcl-xL (~24.5 kDa). In addition, new signals arising from the PUMA^BH3^ domain (Figure 8c,d), are observed in the NMR spectra of the sapC-PUMA/Bcl-xL mixture, whereas sapC signals are minimally perturbed (Figure 8b). These results indicate that the PUMA^BH3^ fragment in the chimera is the binding domain to Bcl-xL.

The spectrum at 0.5:1 molar ratio of Bcl-xL/sapC-PUMA displays two sets of signals for the PUMA^BH3^ domain corresponding to populations of the unbound and bound conformations (signals of the bound conformation indicated by arrows in Figure 8c,d in magenta). These signals are not observed in the spectrum at 0:1 molar ratio (no bound population) and are still observed in the spectrum at 1:1 molar ratio (100% bound population). When the Bcl-xL/sapC-PUMA molar ratio is increased to 1:1 or 2:1, only one set of signals remains, which corresponds to 100% population of bound PUMA^BH3^ (Figure 8c,d). Overall, these results indicate that the sapC-PUMA/Bcl-xL complex is 1:1 and that the exchange rate between the bound and unbound conformations of sapC-PUMA is slow on the NMR chemical shift time scale as two sets of signals are observed.

Because the binding of sapC-PUMA to Bcl-xL is in the NMR slow exchange regime, it is not possible to calculate a K_D_ value (K_D_ = k_off_/k_on_) from the changes in chemical shifts or signal intensity upon binding. The chemical shifts do not represent population averages of the free and bound forms as in the case of fast exchange, and the signal intensities are affected by very different relaxation times of the free and bound species. However, it is possible to estimate an upper limit of the K_D_ value based on the differences in chemical shifts of the two sets of signals. The difference in amide ^1^H chemical shift of PUMA signals in the free and bound forms (~0.082 ppm) (Figure 8c) results in a value for Δω of ~49 Hz at the spectrometer frequency of 601.13 MHz used for these studies. Assuming that binding is diffusion-controlled; k_on_ ~ 10^9^ M^−1^ s^−1^ and k_off_ ~ 10^9^ K_D_ s^−1^. The slow exchange regime observed indicates that k_off_ << 49 s^−1^ and therefore K_D_ << 49 nM, as expected based on previously reported values for the binding of PUMA^BH3^ and Bcl-xL (~3 nM) [16].

We have also checked by NMR that the mutant chimera, sapC-PUMA-DM, binds Bcl-xL with similar upper value of the dissociation constant, as we observe two sets of signals indicative of slow exchange regime in the spectrum of sapC-PUMA-DM upon titration with Bcl-xL (Figure S4, Supplementary Materials).

### 3.5. Liposome Fusion Orchestrated by SapC-PUMA and SapC-PUMA-DM

The liposome fusogenic capabilities of sapC have been reported previously using TEM [7], SEC [7], and dynamic light scattering [8]. We have obtained TEM micrographs of negatively stained liposome samples prepared using different procedures and have observed a large variability of liposome size and significant shape distortion (Figure S5, Supplementary Materials). This result is likely caused by the drying process required for negatively stained TEM image acquisition. We found it difficult to obtain information on the increase of vesicle size upon sapC addition from TEM micrographs of negatively stained liposome samples. We attempted to use size exclusion chromatography (SEC) to monitor the increase in liposome size upon sapC addition, as this is a common method used to determine liposome size distribution and to separate empty from loaded liposomes [33]. However, it has been reported that liposomes interact with SEC matrices in a dynamic and reversible manner, which could result in misleading retention times [34]. To avoid this effect, it is necessary to initially coat the SEC matrix with lipids. We found that consecutive injections of the same liposome preparation in our SEC experiments resulted in chromatograms with peaks at different elution volume (data not shown), thus rendering unreproducible results.

Dynamic light scattering is an ideal technique to investigate liposome size. This technique has been used previously to test liposome fusion mediated by sapC using a protein construct carrying the His-tag [8]. Under the conditions used in our NMR experiments, we found that sapC-PUMA with His-tag tends to aggregate, as indicated by severe line broadening in the ^1^H NMR spectrum when the pH is decreased from 6.8 to 4.2 (conditions necessary for abundant liposome fusion) (Figure S1, Supplementary Materials). In contrast, previously reported NMR studies on sapC without His-tag indicate that the protein is monomeric at acidic pH [9].

**Figure 9:**
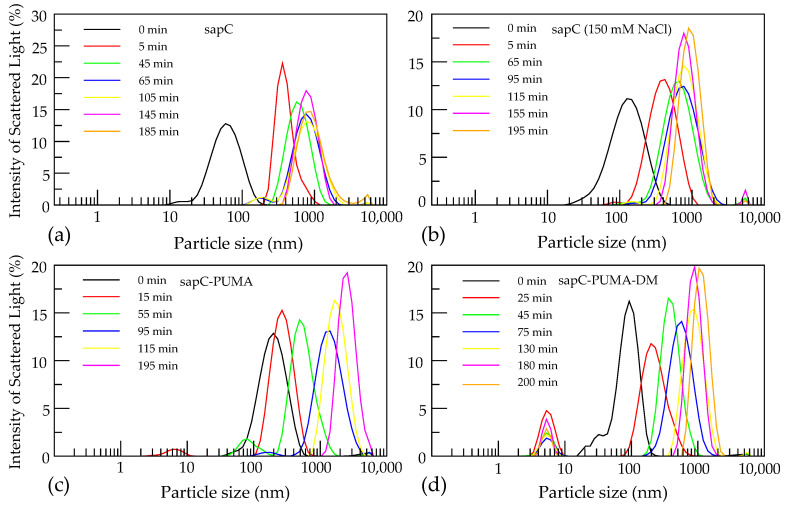
Liposome fusion mediated by sapC, sapC-PUMA, and sapC-PUMA-DM: Analysis of the intensity of the scattered light reveals an increase in liposome diameter (“0 min” indicates liposome size before protein addition) in a time-dependent manner after the addition of (**a**) sapC in water (**b**) sapC in the presence of 150 mM NaCl; (**c**) sapC-PUMA; (**d**) sapC-PUMA-DM. Signals appearing below 10 nm correspond to unbound protein.

We have used dynamic light scattering to study liposome fusion orchestrated by sapC, sapC-PUMA, and sapC-PUMA-DM (all without His-tag) and its time dependence. For sapC, we observe that liposomes increase in diameter from ~100 nm to ~400 nm in approximately 5 min after protein addition and reach approximately 900 nm after 200 min (Figure 9a). After ~65 min, vesicles typically reach maximum size as larger vesicles are not observed at 200 min. It is important to indicate that liposome agglutination could also result in an increase in particle size from dynamic light scattering data. Liposome agglutination would likely result in particles of different size and polydisperse solutions. In contrast, we observed particle size uniformity at the end of the kinetic experiment. In addition, slight liposome leakage was observed in previous studies on liposome fusion mediated by sapC [7,8]. This result is expected for fusogenic processes that require deformation and breakage of the lipid bilayer, but it is not expected from agglutination processes. Thus, our dynamic light scattering results indicate increased liposome size due to fusion and not to agglutination.

We investigated the effect of 150 mM NaCl on liposome fusion mediated by sapC obtaining similar results, which indicates that salt does not play an important role in liposome fusion (Figure 9b). Furthermore, we have determined that both sapC-PUMA and sapC-PUMA-DM are capable of inducing liposome fusion at acidic pH, showing also an approximately 10-fold increase in liposome size in ~200 min after protein addition (Figure 9c,d). These results further prove that the sapC chimeras retain sapC fusogenic function in the presence of the PUMA^BH3^ peptide and non-conservative mutations in sapC. Potentially, this fusogenic effect could be leveraged to design cell penetrating capabilities of biologics based on sapC and sapC mutants.

### 3.6. Cytotoxicity of SapC, SapC-PUMA, and SapC-PUMA-DM in Glioblastoma Cells

Assemblies of sapC with DOPS liposomes have been shown to be highly cytotoxic for a variety of cancer cells and in particular very effective in killing glioblastoma [11,12]. SapC-DOPS is undergoing clinical trial to treat high-grade gliomas [11]. Initial findings indicate that sapC without DOPS liposomes is not cytotoxic [12], thus, it has been suggested that cytotoxicity is a result of a combined effect of sapC bound to liposomes. The mechanism for cell penetration of sapC-DOPS is still not understood and liposome-plasma membrane fusion as well as endocytosis have been suggested as possible mechanisms of entry [11,12,13,14]. Once inside the cell, sapC-DOPS has been reported to trigger apoptosis [12].

Our biophysical studies demonstrate that the sapC chimeras retain the function of both sapC and PUMA at the molecular level. To further test this function, we performed viability assays in glioblastoma cells in the presence of proteoliposomes carrying sapC, sapC-PUMA, and sapC-PUMA-DM with the expectation to observe similar cytotoxicity for sapC proteoliposomes as previously reported [12] and increased cytotoxicity for sapC-PUMA proteoliposomes due to the additional killing effect of the PUMA^BH3^ domain. For this purpose, we followed the preparation of proteoliposomes described in the original assays [12].

**Figure 10:**
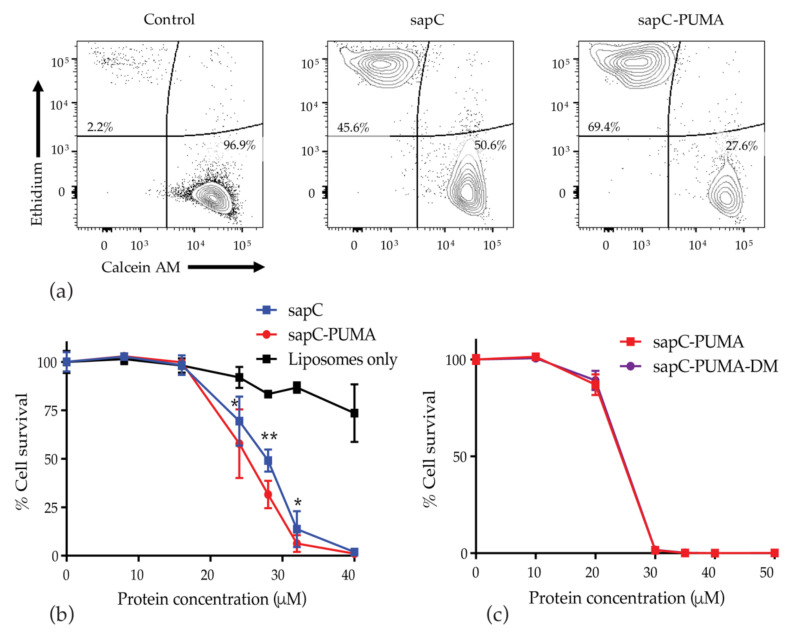
SapC-PUMA proteoliposomes enhance cellular toxicity relative to sapC: U-87 MG cells were cultured for 72 h in the presence of proteoliposomes composed of PS and different concentrations of sapC, sapC-PUMA, and sapC-PUMA-DM, or with liposomes alone (concentrations determined as described in the Materials and Methods section). Cell viability was analyzed by flow cytometry. Calcein AM+Ethidium— live cells from each treatment group were determined and cell survival was calculated relative to PBS control. (**a**) Representative flow cytometry plots of 28 µM protein treatment or PBS control. (**b**) Combined data from all protein concentrations. (**c**) sapC-PUMA and sapC-PUMA-DM proteoliposomes were compared for their effect on cell viability at the indicated concentrations. Data in panels (**b**,**c**) are from different experiments under the same experimental conditions except for slight variations in protein concentration. Data are from at least five replicates per group. Statistical analysis to compare sapC and sapC-PUMA was performed by Student’s *t*-test with * *p* < 0.05; ** *p* < 0.01.

Cell death caused by sapC, sapC-PUMA, and sapC-PUMA-DM proteoliposomes was measured in U-87 MG glioblastoma cells by assessing frequencies of Calcein AM+ live cells and Ethidium+ dead cells by flow cytometry (Figure 10a). Cell death is induced by proteoliposomes starting at ~16 µM protein concentration (Figure 10b). SapC-PUMA shows statistically significant increase in cytotoxicity compared to sapC (Figure 10b), which points to an additive killing effect of the PUMA^BH3^ peptide. A killing activity ratio defined as the percentage of cells killed by sapC-PUMA relative to sapC proteoliposomes, indicates that cytotoxicity is enhanced approximately 40% by sapC-PUMA for certain concentration values (Table 1). These results further indicate that both domains in the chimera are functional, agreeing with the biophysical findings on the dual function at the molecular level. Both sapC and sapC-PUMA induce U-87 MG cell death to nearly 100% at 40 µM protein concentration (Figure 10b). In addition, proteoliposomes composed of sapC-PUMA-DM and sapC-PUMA were compared under the same culture conditions and resulted in very similar cell viability values (Figure 10c, Table 1), indicating that the amino acid substitutions did not impact the functionality of the engineered protein. It is important to note that the mutations in sapC-PUMA-DM are designed to potentially improve delivery by enhancing liposome binding and not to alter the killing effect of the native chimera. Thus, similar killing activity is expected for sapC-PUMA and sapC-PUMA-DM. In contrast to the proteoliposomes, the isolated proteins (i.e., in the absence of liposomes) were not cytotoxic (data not shown) and liposome preparations in the absence of protein were only slightly cytotoxic (Figure 10b) as previously observed for DOPS liposomes [12]. In addition, proteoliposomes of sapC and sapC-PUMA were tested in primary human astrocytes; no significant enhancement in killing activity was observed for sapC-PUMA proteoliposomes relative to sapC at all concentrations (Table 1).

## 4. Discussion

We show by NMR that both domains (sapC and PUMA^BH3^) in the chimera are still functional at the molecular level and that the engineered proteins show, as expected, increased cytotoxicity due to an additive effect of the PUMA peptide. SapC-PUMA retains the saposin fold and sapC functionality by exhibiting reversible pH-dependent binding to liposomes and liposome fusogenic capability. In addition, the PUMA^BH3^ component of the chimera binds tightly to Bcl-xL, its natural antagonist, in the presence of the sapC domain. These results indicate that both sapC and PUMA^BH3^ have minimal interference in each other’s function, thus fulfilling the basic requirement of the chimera design.

Previous work reported that maximum binding of sapC to liposomes occurs upon acidification [9]. Here we show that binding can be observed and quantified at mildly acidic conditions (pH 6) by increasing the lipid to protein molar ratio. Importantly, we demonstrate that it is possible to modify the affinity of the protein to liposomes by changing the overall charge of sapC’s electrostatic surface by non-conservative mutations. These modifications serve a two-fold purpose: (1) increase the overall solubility of the chimera while retaining the saposin fold, and (2) tune liposome-binding dependence with pH. Liposome-binding affinity of the chimera is almost doubled by replacing two acidic for basic amino acids. This result can be of particular relevance for the known biotechnological applications of saposin proteins and lipid assemblies as natural mimics of the lipid bilayer to study integral membrane proteins [35,36]. In addition, the significant increase in liposome binding of the chimeric mutant at pH 7 points to the possibility of keeping sapC chimeras bound to the liposome outer leaflet at physiological pH for different biomedical purposes. Our results on the possibility to pH-tune liposome binding by mutating sapC open the door to potentially target tissues at different pH values, analogously to current research efforts directed to target tumors and control drug release at different pH values by proper liposome functionalization [37].

By performing biochemical and biophysical studies, we have determined that both domains of the chimera protein retain proper fold and binding capabilities, which gives greater confidence to the effectiveness of the chimera constructs prior to performing in vitro cell studies. Importantly, our viability studies indicate increased cytotoxicity for sapC-PUMA compared to sapC proteoliposomes. This is a promising result showing that potential anticancer peptides can be added to sapC to elicit additional cytotoxicity. Because sapC-DOPS is currently on clinical trial to treat refractory glioblastoma [11], an increase in cytotoxicity close to 40% could result in beneficial effects.

Altogether, our results indicate that sapC is a good candidate for engineering chimeric biologics by adding peptides or small proteins as potential therapeutics. Future work is necessary to test the effect of other therapeutic peptides/proteins in sapC chimeras at the molecular and functional levels in the presence and absence of liposomes or lipid nanoparticles, and to further tune liposome binding affinity with additional mutations to target specific tissues and promote liposome fusion under different pH conditions.

## Figures and Tables

**Table 1:** Killing activity of sapC-PUMA and sapC-PUMA-DM proteoliposomes: killing activity is defined as the ratio of percentages of cells killed by the indicated protein treatments from assays as described in Figure 10. A ratio of 1 indicates equal killing between the specified proteins at a given concentration. Underlined values denote enhanced killing activity with a *p*-value < 0.05 by Student’s *t*-test. Data are from at least five replicates per group.

U87-MG				Human Astrocytes	
SapC-PUMA/SapC		SapC-PUMA-DM/SapC-PUMA		SapC-PUMA/SapC	
Conc. (µM)	Killing Activity	Conc. (µM)	Killing Activity	Conc. (µM)	Killing Activity
24	1.38	20	0.83	20	0.97
28	1.35	30	0.99	30	1.04
32	1.09	35	0.99	35	0.99
40	1.01	40	1.00	40	1.02

## Data Availability

NMR chemical shifts of saposin C used in this study are openly available in the Biological Magnetic Resonance Bank at doi:10.13018/BMR5465, reference number 5465.

## Supplementary Materials

The following are available online at https://www.mdpi.com/article/10.3390/pharmaceutics13040583/s1, Figure S1: SapC-PUMA with His-tag aggregates upon acidification; Figure S2: Double-mutant sapC-PUMA-DM shares structure identity to sapC-PUMA; Figure S3: Increased binding of sapC-PUMA-DM to liposomes; Figure S4: The PUMA region in sapC-PUMA-DM also binds Bcl-xL with high affinity; Figure S5: Liposome shape and size distortion in negative stained samples for TEM analysis.
