# Supplementary material for: Engineering of Saposin C Protein Chimeras for Enhanced Cytotoxicity and Optimized Liposome Binding Capability
Source: Pharmaceutics. 2021 Apr 19;13(4):583. doi: 10.3390/pharmaceutics13040583 (PMC8072984; doi:10.3390/pharmaceutics13040583)
Supplement: Supplementary file 1 [file pharmaceutics-13-00583-s001.zip › pharmaceutics-1152516-supplementary.pdf]

pharmaceutics

Supplementary Materials: Engineering of Saposin C Protein Chimeras for Enhanced Cytotoxicity and Optimized Liposome Binding Capability

Suzanne I. Sandin, David M. Gravano, Christopher J. Randolph, Meenakshi Sharma and Eva de Alba

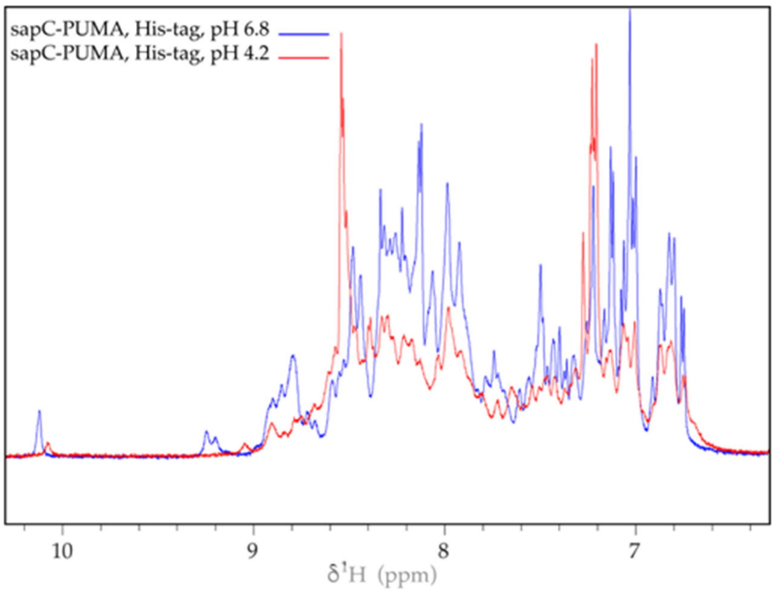

Figure S1. SapC-PUMA with His-tag aggregates upon acidification: SapC-PUMA with His-tag at pH 6.8 (blue, overall intensity from integration set to 100%) and pH 4.2 (red, intensity of 77% compared to the spectrum at pH 6.8).

Citation: Sandin, S.I.; Gravano, D.M.; Randolph, C.J.; Sharma, M.; de Alba, E. Engineering of Saposin C Protein Chimeras for Enhanced Cytotoxicity and Optimized Liposome Binding Capability. *Pharmaceutics* **2021**, *13*, 583. https://doi.org/10.3390/pharmaceutics13040583

Academic Editor: Anna Angela Barba

Received: 4 March 2021
Accepted: 14 April 2021
Published: 19 April 2021

Publisher's Note: MDPI stays neutral with regard to jurisdictional claims in published maps and institutional affiliations.

Copyright: © 2021 by the authors. Licensee MDPI, Basel, Switzerland. This article is an open access article distributed under the terms and conditions of the Creative Commons Attribution (CC BY) license (http://creativecommons.org/licenses/by/4.0/).

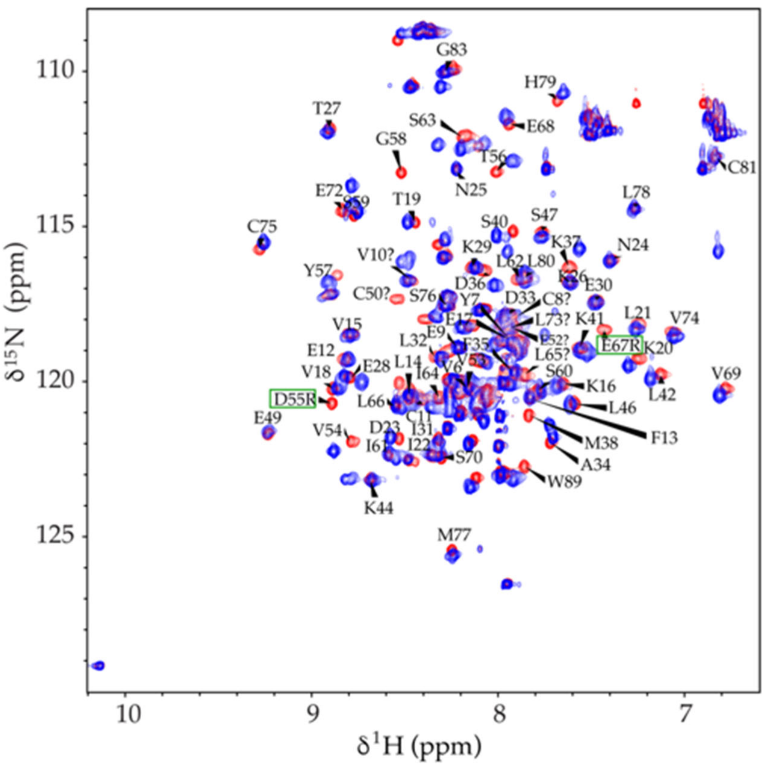

Figure S2. Double-mutant sapC-PUMA-DM shares structure identity to sapC-PUMA: [^{1}H,^{15}N]-sofast HMQC of sapC-PUMA (blue) and sapC-PUMA-DM (red) at pH 6.8 The amino acids that are mutated are shown with green rectangles. Other assignments are shown with the corresponding labels.

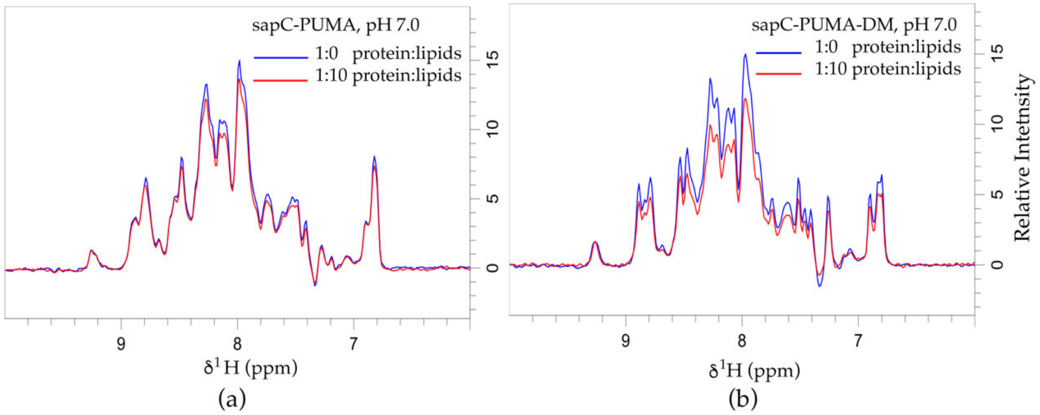

Figure S3. Increased binding of sapC-PUMA-DM to liposomes: 1D projections of [^{1}H,^{15}N]-sofast HMQC of sapC-PUMA (**a**) and sapC-PUMA-DM (**b**) in the absence (blue) and presence (red) of lipids at 1:10 protein:lipid molar ratio, pH 7. The binding of sapC-PUMA-DM is significantly larger compared to the wild-type chimera (8.5% vs. 28%) according to the decrease in signal intensity resulting from liposome binding.

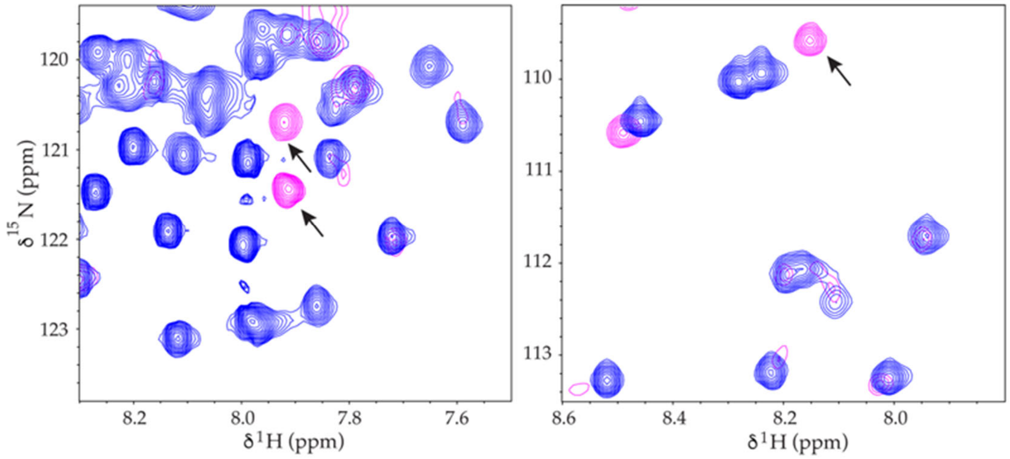

Figure S4. The PUMA region in sapC-PUMA-DM also binds Bcl-xL with high affinity: Selected regions of [^{1}H,^{15}N]-sofast HMQC spectra of sapC-PUMA-DM in the absence (blue) and in the presence (magenta) of 1:1 molar ratio of unlabeled Bcl-xL. The regions shown are equivalent to those appearing in Figure 8 c,d.

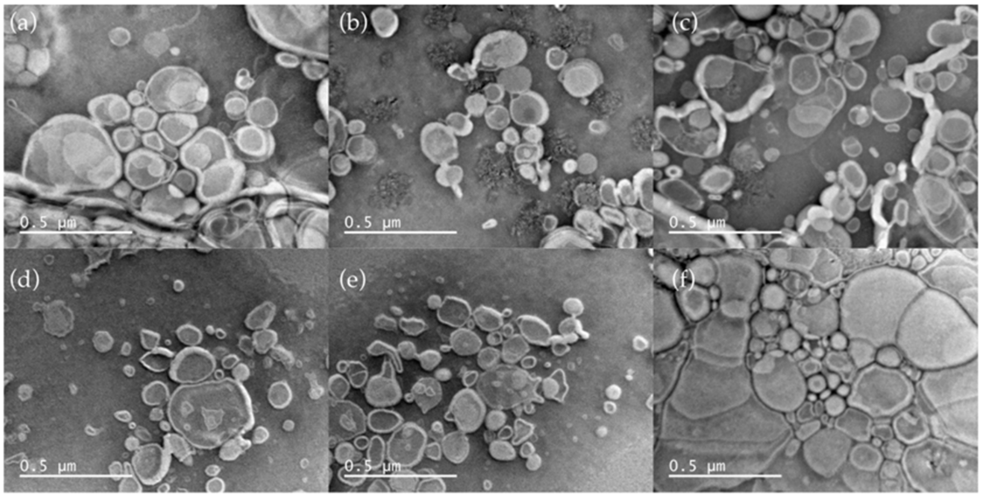

Figure S5. Liposome shape and size distortion in negative stained samples for TEM analysis: Micrographs (**a**)–(**c**) (same liposome sample) and (**f**) (different liposome sample but identical preparation procedure as (**a**)–(**c**)) were acquired after allowing the liposome solution to dry before staining. Micrographs (**d**,**e**) (same liposome samples as (**f**)) were allowed to dry for 20 min.
